# Dynamic stress characterization and instability risk identification using multisource acoustic signals in cut-and-fill stopes

**DOI:** 10.1038/s41598-024-66445-8

**Published:** 2024-07-17

**Authors:** Longjun Dong, Yihan Zhang, Zhongjie Chen, Yongyuan Kou, Zhongwei Pei

**Affiliations:** 1 The School of Resources and Safety Engineering, Central South University, Changsha, 410083 China; 2 Jinchuan No.2 Mine, Jinchuan Group Co., LTD., Jinchang, 737100 China; 3 School of Civil and Resource Engineering, University of Science and Technology Beijing, Beijing, 100083 China

**Keywords:** Microseisms, Cut-and-fill mining, Stress redistribution, Risk identification, Velocity tomography, Spatial b-value, Geophysics, Petrology, Civil engineering

## Abstract

The quantitative characterization of rock mass and stress changes induced by mining activities is crucial for structural stability monitoring and disaster early warning. This paper investigates the time–space–intensity distribution of microseismic sources during the pillar-free large-area continuous extraction. Furthermore, it explores a method involving collaborative evolution patterns of the velocity field and spatial *b*-value to identify stress and structural changes at the panel stope. Results show that anomalous zones in wave velocities and *b*-values form at the intersections of extraction drifts, strike drifts, cross drifts, and connection roadways influenced by mining activities, as well as in footwall ore-rock contacts, often accompanied by the nucleation of microseismic events. The synergistic use of wave velocity fields and spatial *b*-value models reveals the relationship between stress migration behavior and stope structure changes due to mining disturbances. The velocity field primarily reflects macroscopic changes in the structure and stress distribution, while spatial *b*-values further explain stress gradients in specific areas. Additionally, we have advanced the identification of an instability disaster at the connection roadway and cross drift intersection based on increases in wave velocity and abnormal changes in *b*-value. This paper demonstrates the potential of risk identification using the proposed method, providing insights into predicting geotechnical engineering disasters in complex stress environments.

## Introduction

The increasing requirements of mineral resource production and mining operations have escalated mine production safety issues due to the stress induced by mining activities^1–3^. This is particularly pronounced in mining environments with multiple segments and stopes, with complex relationships among ore bodies, surrounding rock, voids, and backfilled regions^4,5^. In high-frequency extraction and backfilling processes, production roadways intersect with cutting entries, and the spatial layouts of the mining faces constantly change, leading to frequent and exceptionally complex spatiotemporal stress distributions. The concentration and relaxation of stresses in structural components commonly lead to incidents such as roof collapses, sidewall failures, and even rockbursts and strong tremors^6,7,9^, causing casualties and vehicle damages^10,12,13^. Hence, the dynamic characterization of stress states and the early identification of high-risk disaster-prone areas are crucial to address and reduce stope production accidents and optimize mining plans^14–16^.

Currently, researchers have adopted various methods to monitor the deformations and movements of rock masses and backfilled areas, including deformation measurement instruments^14,17^, numerical simulation methods^18–20^, and physical model tests^21–26^. However, these methods have limitations. For example, traditional deformation measurement instruments can monitor only local positions; thus, the overall condition of the mining area cannot be comprehensively understood. Numerical simulation methods are limited by the accuracy and complexity of the parameters, resulting in limited predictive effects in practical engineering applications. Physical model tests are limited by size- and time-related scaling problems, preventing complete simulations of the actual working conditions of extraction and backfill areas. Microseismic monitoring technology^27^ is an acoustic monitoring technique that utilizes various signals, such as those generated by rock fractures, deformations, and rock drilling blasts^28–30^. In this method, source parameters and seismic activities^31–34^ are statistically analyzed to monitor rock deformation and movement within mines in real time. However, the application of this technology at the scale of mining panels has mainly focused on longwall coal mining in coal mines^35,36^. Compared to coal mining, thick and large precious metal mines using the downward cut-and-fill method with cemented backfill also exhibit large-scale and continuous mining production characteristics. However, significant differences exist in rock conditions, operational procedures, and waveform characteristics within the panel. These differences make it challenging to directly apply the microseismic monitoring design approaches from coal mining or other metal mines. For instance, sensors need to be installed within the backfill roof of previously mined layers, with each layer being approximately 4 m apart. Due to this sensor arrangement, signals from blasting, drilling, and microseismic sources have short propagation distances and low attenuation, leading to a higher proportion of high-frequency noise during acquisition. This not only affects the accuracy of arrival time picking and waveform feature extraction but also makes it difficult to distinguish blast signals from adjacent production drifts. Therefore, microseismic monitoring technology needs adjustments and optimizations in layout designs, signal processing methods, and data analysis techniques to address issues such as high-frequency noise resistance and waveform separation.

In recent years, acoustic tomography has been proposed as a technique for inferring the high-stress distribution areas in underground mines using acoustic signals collected by microseismic monitoring systems^37^. This approach is simple, quick, and cost-effective^38^. Acoustic tomography has been utilized to image the locations of stress concentration zones, anomalous geological structures, and areas with high seismic activity during coal mining operations^39^. Acoustic tomography is a powerful method for quantifying seismic activity and providing valuable information for dynamic hazard assessment in mines. Due to the complex rock mass conditions and mining technology used in stopes, the imaging network layout and ray distribution are both limited, which greatly restricts the application of tomography techniques in mines at the stope scale^40–42^. Thus, its application in complex environments such as metal ore mining fields, where the distribution of backfill materials, ore bodies, and rock formations is intricate, remains rare.

It is imperative to address the technical challenge of how to monitor the changing stress environment during cyclical operations such as excavation, blasting, and backfilling to ensure the safety of mining operations. This study proposes utilizing multiple acoustic techniques to monitor stress variations within complex mining structures under dynamic disturbances to identify potential high-risk disaster-prone areas in advance. The multisource acoustic techniques utilized in the Jinchuan mining field are determined based on the rock mass properties and mining procedures after the monitoring range and network layout are established. Passive signals related to rock mass damage and deformation, such as microseismic events, and active signals generated by drilling and blasting operations are analyzed, and the localization, spatial *b*-value scanning, and velocity field results are used to analyze stress transfer and variation within the mining field in real time. Additionally, high-risk areas within the stope are identified, and guidance is provided for the mining process.

## Methods

### Conception of multisource acoustic signal monitoring

The rock composition, engineering structure, and geostress environment in the mining area are complex, and frequent mining operations intensify stress redistribution and hazard zone variations. Limited on-site microseismic data and single physical characterization methods make stress representation and rock instability risk identification difficult. Among the numerous acoustic sources distributed in the mining area, microseismic signals not only carry the source characteristics in deformation and fracture of rock and backfill but also, along with the acoustic waves generated by production activities, collectively store the structural and stress information of the mining field during the propagation process in the medium. Specifically, passive sources, mainly including microseismic signals, are advantageous for monitoring continuous stress changes and assessing rockburst risks in mining areas. However, relying solely on passive microseismic signals fails to accurately identify the stress distribution and associated instability risks during excavation, extraction, and backfilling^35,43^, because velocity field imaging depends on a uniform distribution of rays. On the other hand, active sources, which mainly include signals generated by rock drilling, impacts, and explosions, are beneficial for the advanced detection of stress distributions and geological structures during excavation operations. Yet, the exclusive use of active signals generated by mining operations limits the temporal and spatial scales of the signals, thus hindering continuous monitoring of stress distribution beyond the excavation area. Therefore, based on the waveform identification and source localization of multi-source acoustic signals in the mining area, a complementary distribution of P-wave rays is established by considering the accurate coordinates of various production sources and the widespread distribution of microseismic sources. This approach effectively enhances the accuracy of velocity field imaging using passive signals. Given the concentrated production scale of the panel, the generated microseismic signals are often closely related to deformation and rupture processes during mining operations and the effects of adjacent rock formations. Furthermore, by correlating the seismic velocity field, microseismic activity, and internal stress distribution^38,44^, the combined approach dynamically characterizes stress and identifies instability risks in cut-and-fill stopes.

### Experimental site and monitoring system

**Figure 1:**
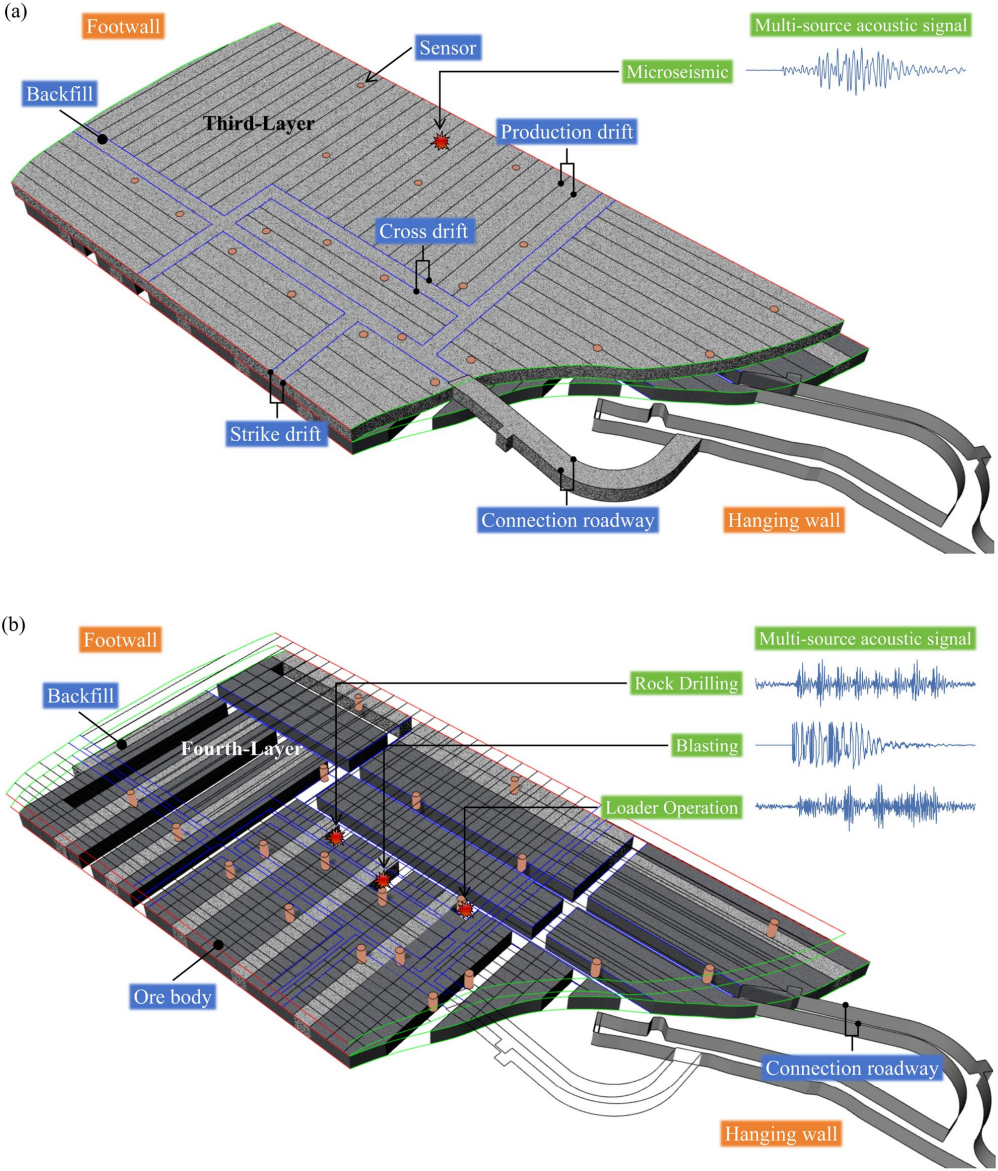
Cemented horizontal undercut-and-fill mining in stopes, the sensor arrangement, and the collected multisource geoacoustic signals. (**a**) 3rd layer of the VI panel stope, (**b**) 4th layer of the VI panel stope (production status in December 2023).

In December 2022, a multisource acoustic and microseismic monitoring system was established in the VI panel stope at the 1018 m sublevel in Jinchuan Second Mine Area. This panel stope, which has an annual production capacity of nearly 600,000 tons out of the mine’s total 4.5 million tons, reaches mining depths of up to 800 m. The area spans 120 m and 183 m on either side of the vein, with a width of 94 m, characterized by complex mining conditions such as high ground pressure, robust rock strength but fragmented rock masses and surrounding rock. Additionally, the horizontal stress exceeds the vertical stress. A relatively safe but high-cost, deep mechanized downward stratified horizontal stope cemented filling mining method is employed here, suitable for extracting valuable metals in non-self-supporting, highly fragmented, and stressed goaf areas. Panel mining is conducted in layers with a 4.3 m interval, and Fig. 1 displays the structures of the 3rd and 4th layers during the mining process of the 4th layer.

**Figure 2:**
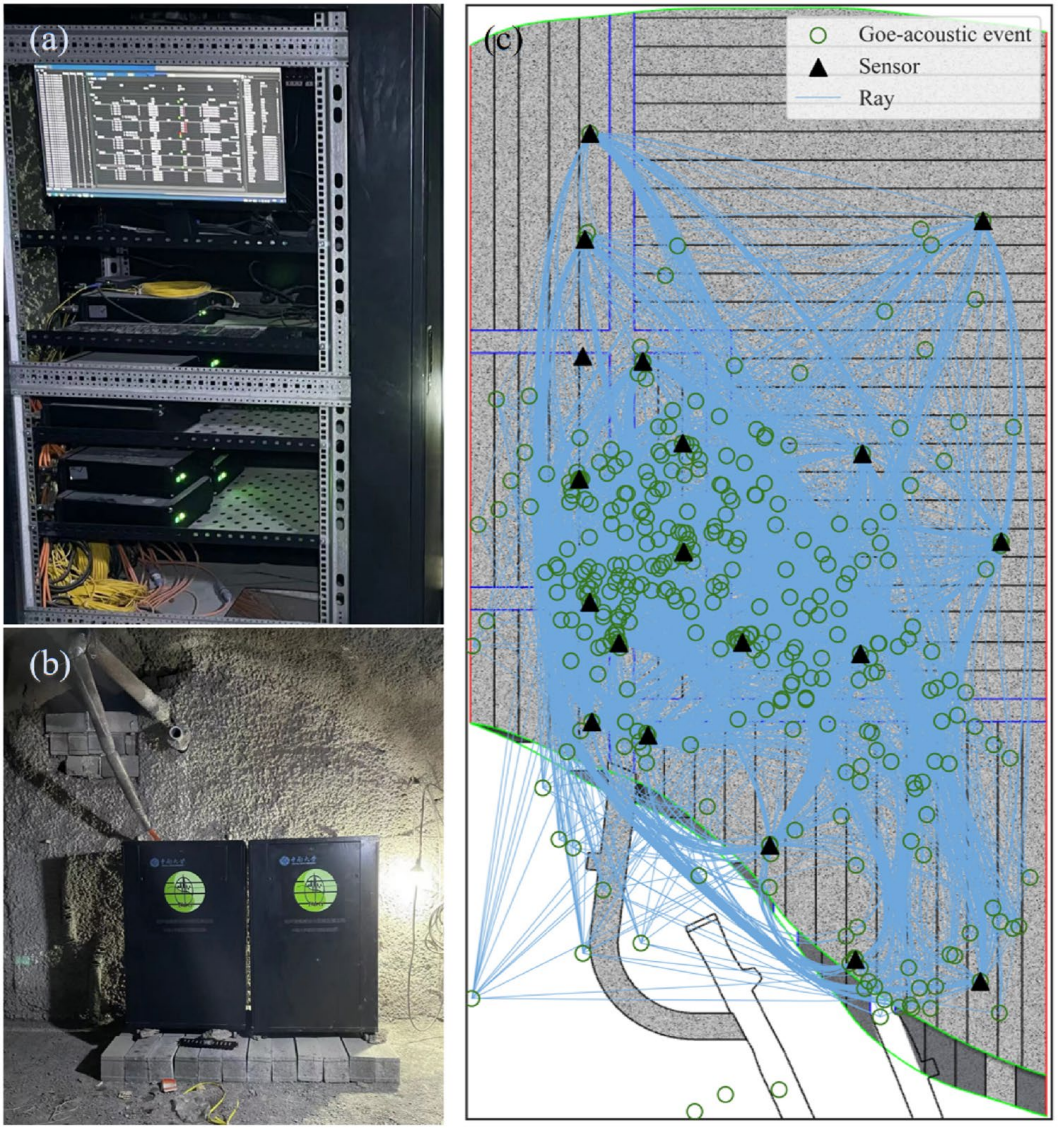
(**a**) Monitoring system; (**b**) storage chamber; (**c**) P-wave ray paths of events that occurred from March 3, 2023, to March 11, 2023.

The installed sensors, with a sensitivity of 30 V/g and a frequency response ranging from 2 Hz to 2 kHz, are installed in the mining drifts of the 3rd layer to monitor microseismic activity during production in the 4th layer. The arrangement of sensors and cables follows the mining sequence, being placed from the footwall to the hanging wall of the ore body. Subsequently, the cable lines are gradually positioned along the corss drifts and strike drifts, with both the sensors and cable lines left within the fill body. These cables are connected to the data collection system located in the monitoring chamber within the connection roadway of the 3rd layer. The monitoring system and the monitoring chamber are depicted in Fig. 2a,b, respectively.

Due to frequent blasting and roof settlement in the backfilled stope, the sensor cables originally deployed near the footwall were damaged, preventing the sensor monitoring network from effectively collecting signals in this area. This analysis uses data collected in March 2023, during which mining activities were mainly concentrated in the central and upper areas of the panel. The number of sensors in these areas meets the monitoring and analysis requirements. Additionally, a data transmission failure from March 11 to 16 led to some data loss. As shown in Fig. 2c, the distribution of multisource acoustic event rays within the sensor monitoring network effectively covered the analysis area.

### Data cleaning and source location

The acoustic signals used in the data analysis were generated by various sources, such as blasting caused by extraction operations, drilling with drilling jumbo equipment, operations performed with load-haul-dump equipment, and microseismic signals generated by rock mass and fill body fracture and deformation processes (Fig. 1). However, acoustic signals generated by activities such as personnel and vehicle movements typically trigger less sensors and have lower signal-to-noise ratios than the other signals, resulting in less accurate P-wave arrival times and reduced localization accuracy. Therefore, in the data cleaning process, all events were manually classified, and only events that activated five or more sensors were retained, while events with lower signal-to-noise ratio signals were removed.

The multisource acoustic event signals were processed with the wavelet-STA/LTA-AIC picker method^8^ to identify waveform arrival times, and the LM localization method^11^ was utilized to determine the coordinates of the sources. The localization accuracy was calibrated and verified through tapping and blast experiments in the panel mining area. Tapping was randomly conducted 44 times in the 4th layer of the stope, and the localization errors of the tapping sources were calculated. The total localization error in the x and y directions was 5.89 (2.73, 7.54) meters, with an average localization error of 6.21 m. The average error for the 11 sets of blast sources was 3.15 m. This meets the requirements for velocity field imaging and analysis of the spatiotemporal distribution of the seismic sources.

**Figure 3:**
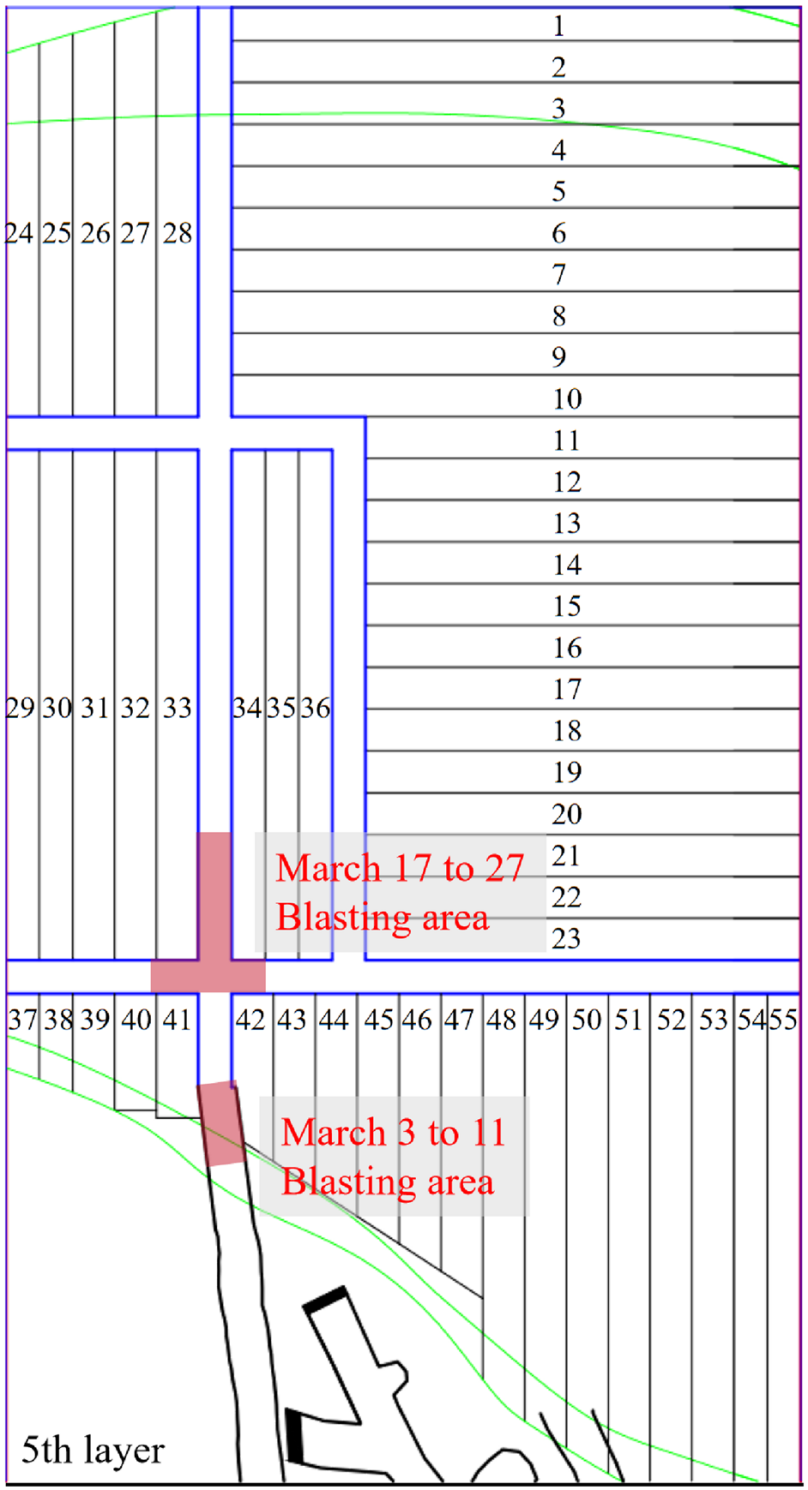
Blasting mining area in the 5th layer of the VI panel stope.

Notably, due to the large number of sensors included in the 3rd layer, the monitoring network has an approximately horizontal distribution. Therefore, the seismic source location encounters challenges in effectively constraining the coordinates in the z-direction, resulting in localization errors typically reaching around 10 m in the z-direction. However, as shown in Fig. 3, tunnels were being excavated with localized blasting activities in in the 5th layer in the panel stope in March, while no mining activity occurred at other vertical heights within the VI panel stope. It can be assumed that almost all microseismic signals originate from the stoping operations in the fourth layer and the area affected by mining activities in the 3rd layer. Therefore, the vertical (z-direction) location error does not affect the analysis of the seismic source activity within the mining area.

### Spatial *b*-value scanning

In the least squares method, the *b*-value is calculated based on the G-R relationship. Thus, there is often a large error in processing large-magnitude event data, for which less data is typically available. The maximum likelihood estimation method assigns equal weights to each microseismic event and requires fewer data samples for calculations. After comparing the performance of the two methods, the maximum likelihood estimation method was used to calculate the *b*-values. The formula is as follows:

1 $$b = \frac{n\lg e}{{\sum\limits_{i = 1}^{n} {(M_{i} - M_{0} )} }}.$$

In the equation, $n$ represents the number of microseismic events, $M_{i}$ represents the magnitude of each microseismic event, and $M_{0}$ represents the magnitude of the initial event. The equation can be rewritten as:

2 $$b = \frac{\lg e}{{\overline{M} - M_{c} }}.$$

In the formula, $M_{c}$ is the minimum complete magnitude within the statistical region, and $\overline{M}$ is the average magnitude of microseismic events with magnitudes greater than $M_{c}$.

In the *b*-value spatial scanning calculations, the mining area is divided into a 50 × 95 grid with a side length of 2 m. Furthermore, each grid node is taken as the center of a circular region with a radius of 10 m as the statistical unit for event scanning. First, $M_{c}$ is calculated for each unit, and then the *b*-value of the microseismic event is calculated. The maximum location error in the localization accuracy verification test was 12.26 m, and 90% of the tapping sources had location errors within 10 m. Therefore, a scanning radius of 10 m was determined for the statistical unit to ensure the validity of the searched seismic source locations within the node search range. Circular statistical units with at least 40 microseismic events were included in the calculations, and the remaining areas were represented as empty regions.

### Wave velocity field tomography

Tomography techniques involve two processes: forward and inverse. In the forward process, a grid is established in the mining field model, and ray paths from sources to sensors are computed based on the velocity field. In a nonuniform velocity field, ray paths may experience diffraction. The sparse ray path matrix and the arrival time data received by the sensors are computed in this process. This study uses the fast sweeping method (FSM) for the forward approach. FSM calculates arrivals of regional nodes using a Gauss–Seidel iterative scanning approach, setting the arrival time of source node to zero and other nodes to extreme values initially. The Gauss–Seidel iterative algorithm then solves the eikonal equation, retaining the minimum value between the original and updated values until convergence. FSM offers higher computing efficiency than the fast marching method (FMM), making the forward calculation more efficient and significantly improving overall tomography computation.

The inversion process can be represented as the solution to an optimization problem $f$ considering the observed arrival time ${d}_{obs}$ and computed arrival time ${d}_{cal}$, as shown below:

3 $$f={\Vert {d}_{obs}-{d}_{cal}\left({m}_{i+1}\right)\Vert }^{2},$$

4 $${m}_{i+1}={m}_{i}+dm,$$

5 $${d}_{obs}-{d}_{cal}\left({m}_{i}\right)=Ldm,$$

where $dm$ is the update of the slowness model. Since the misfit between the observed and calculated arrivals primarily arises from variations in ray paths through different slowness fields, the prior slowness field can be optimized by minimizing the misfit function to approach the actual slowness field. Typically, the inversion problem is characterized as either underdetermined (more voxels than rays) or overdetermined (more rays than voxels). In this study, the DLSQR method is used due to its good convergence speed and reliability in solving ill-posed linear equations. This method has a low computational cost as it does not require calculating a gradient matrix, simplifying computations. Only the nonzero elements of the large sparse matrix need to be calculated, reducing the storage requirements and computational cost.

## Results

### Spatial–temporal characteristics of microseismic events

**Figure 4:**
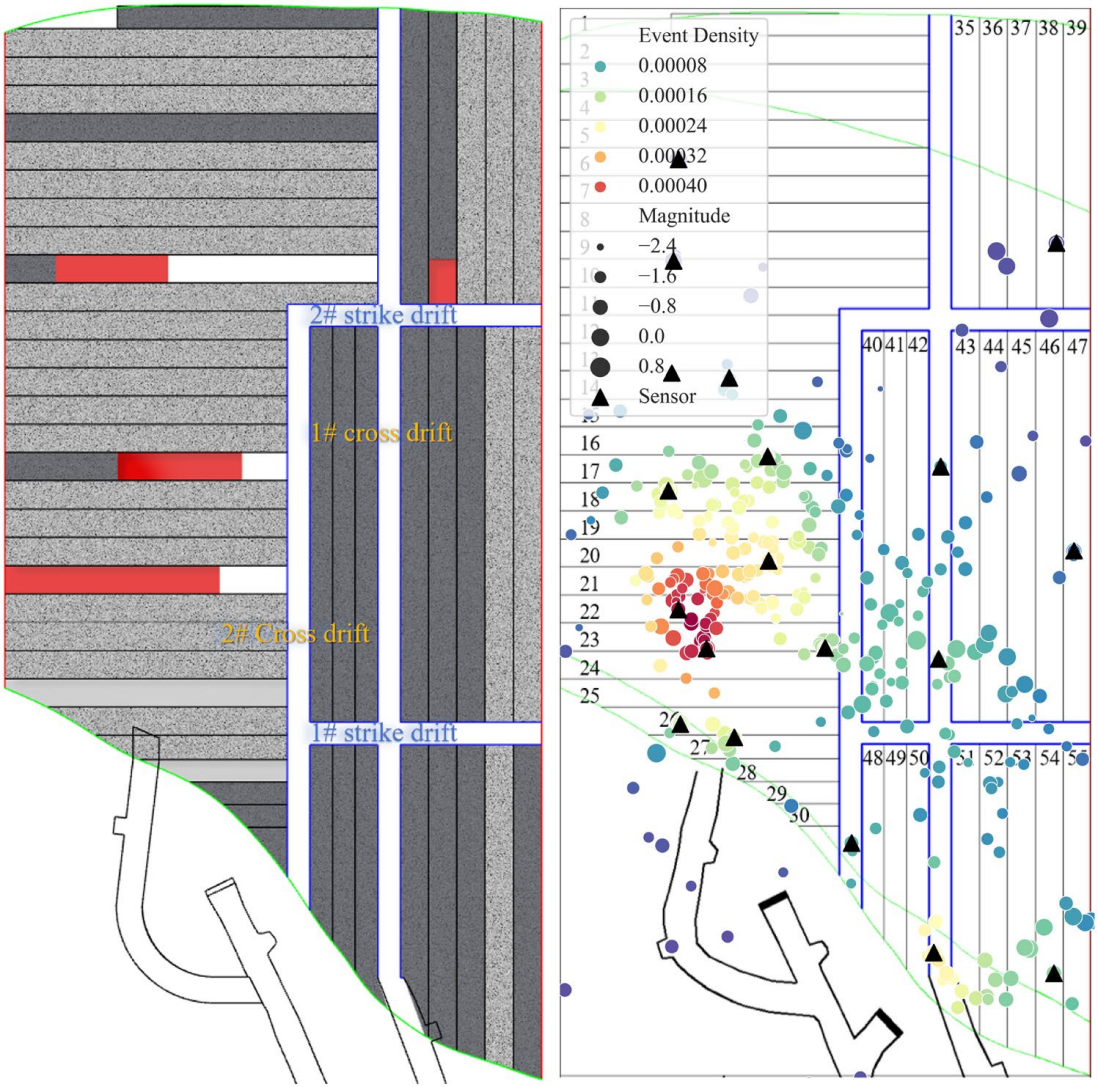
Mining status and seismic activity in the VI panel stope of the 1018 m sublevel from March 3, 2023, to March 11, 2023: (**a**) production overview (deep brown represents the ore body, gray represents the backfill body, red represents the blasting area in the mining drifts, white represents the strike drifts, cross drift, and mining drifts, light gray represents recent backfilling drifts with a curing time of no more than 28 days); (**b**) event location, density, and magnitude.

The mining operation performed in the 4th layer of the VI panel stope in the 1018 m sublevel from March 3, 2023 to March 11, 2023 is shown in Fig. 4a. During this period, the mining and backfilling activities were primarily concentrated on the left side of the 2# cross drift. Recent backfilling operations were completed for the 2 weeks ago, while backfilling operations were performed in the 25# and 28# drifts. Explosive blasting operations were carried out in the 10#, 17#, 21#, and 36# drifts. The drifts in which backfilling operations were recently completed can be approximated as void spaces. This is because, within a short period, the backfill material has not yet provided adequate support, and the fill body roof is still exposed. The difference is that the drifts undergoing backfilling operations have a longer roof exposure time during the corresponding period.

Figure 4b presents the distribution of the microseismic event density and intensity versus the spatial structure of the mining area over this period. Two distinct nucleation areas formed inside the panel stope, and some microseismic events with magnitudes exceeding 0 occurred within these nucleation areas. These two nucleation areas are located adjacent to the 17# and 21# drifts and the intersection area between the ore-rock transition zone and the layered connection roadway near the hanging wall. The other seismic sources are distributed along the strike drift and cross drift as well as on both sides of the backfilling drifts. In particular, seismic activity occurs frequently near the 2# cross drift, indicating that when the semipermanent drift within the exposed roof occurs close to the extraction drifts, obvious microseismic activity is generated due to the impact of blasting disturbances and excavation. In addition, there are a few seismic sources around the 10# and 36# drifts. However, due to the limited number of sensors and the minimum triggering thresholds for microseismic events near the 10# and 36# drifts, fewer microseismic events with higher magnitudes are recorded in the areas adjacent to these two drifts.

**Figure 5:**
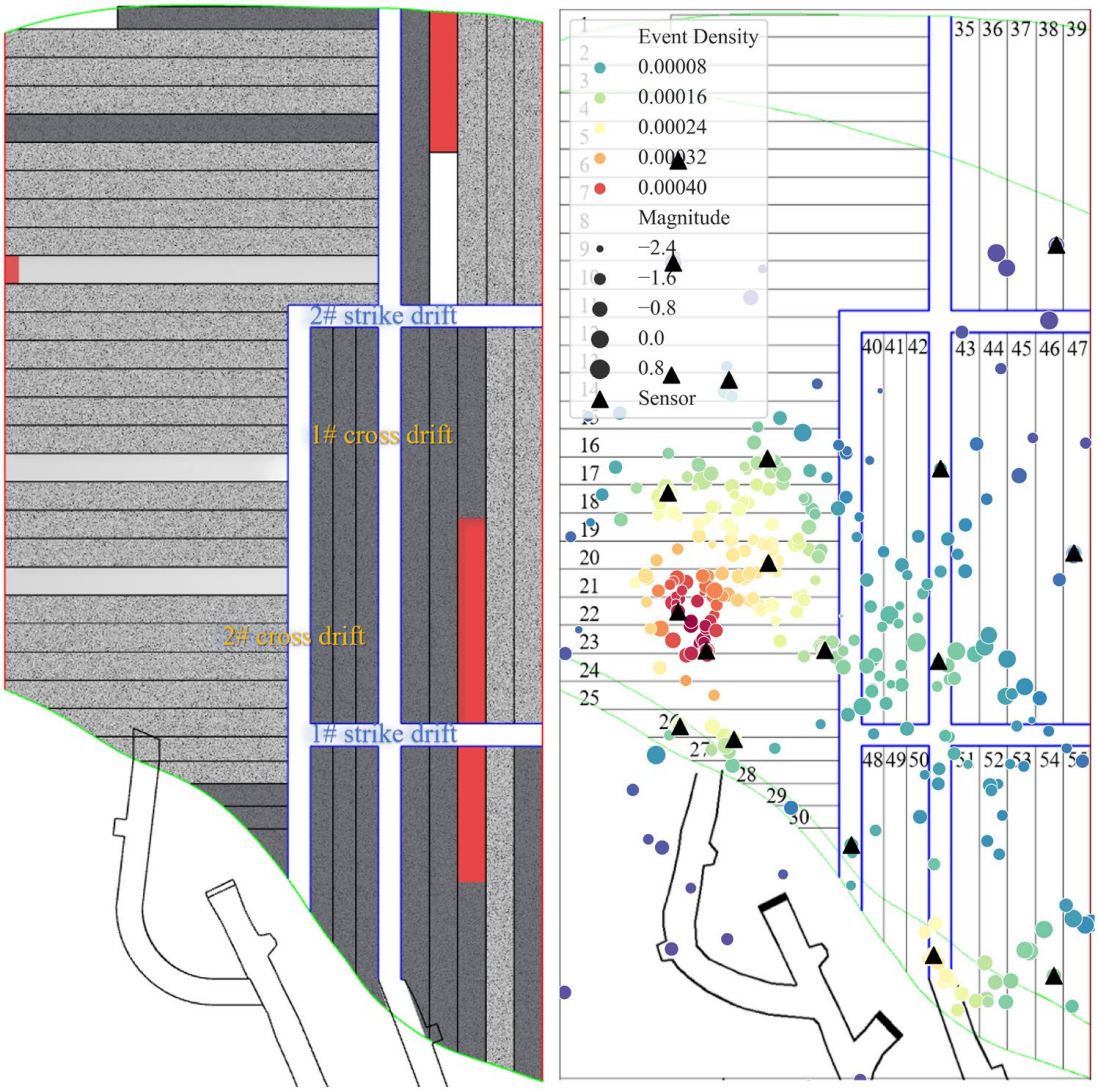
Mining status and seismic activity in the VI panel stope of the 1018 m sublevel from March 17, 2023, to March 27, 2023: (**a**) Production overview (deep brown represents the ore body, gray represents the backfill body, red represents the blasting area in mining drifts, white represents the strike drifts, cross drift, and mining drifts, light gray represents recent backfilling drifts with a curing time of no more than 28 days); (**b**) event location, density, and magnitude.

As shown in Fig. 5a, by middle and late March, the blasting mining drifts on the left side of the 2# cross drift were all completed and backfilled after the mining operations were completed, and the production area shifted to one side of the 1# strike drift. During this period, blasting operations were conducted in the 10#, 36#, 45#, and 53# drifts, while backfilling operations were carried out in the 10#, 17#, and 21# drifts..

Compared to early March, the density and intensity of microseismic events changed significantly in three areas by late March (Fig. 5b). First, the number and magnitude of the seismic sources between the 17# and 21# drifts both decreased. However, numerous microseismic events still occur, and these seismic sources are spatially closer to the direction of the blasting face. In the short period after the mining operations were completed, the roof of the drifts near the blasting face showed stress release characteristics to some extent. Second, the prominent seismic source nucleation areas are vertically distributed in the central sections of the 21# and 25# drifts. The area in the 5th layer in which cutting preparation work was performed (Fig. 3) and the newly formed nucleation area correspond in the horizontal direction. Thus, the blasting operations in the strike drift and cross drift led to significant seismic activity. Third, the intensity and density of seismic source activity within the nucleation area at the intersection of the connection roadway increased. In addition, most events with high magnitudes occurred in the actively mined 36#, 45#, and 53# drifts, as well as on both sides of the strike drifts. Notable seismic activity was also observed at the intersection of the 1# strike drift and the 1# cross drift due to the blasting activities in the 45# and 53# drifts, while the seismic activity near the 2# cross drift was reduced due to the completion of the mining operations in the drifts on the left side.

### The evolution of the velocity field

The tomographic technique utilizes the difference in arrival times resulting from variations in acoustic wave propagation paths within a rock mass to infer the wave velocity structure inside the rock. The velocity of the rock mass is related to both its internal structure and its stress state. By reconstructing the velocity structure within the mining area using tomographic techniques, the stress distribution within the advancing face can be monitored and predicted during excavation processes. Moreover, the interface condition can be investigated after backfilling.

**Figure 6:**
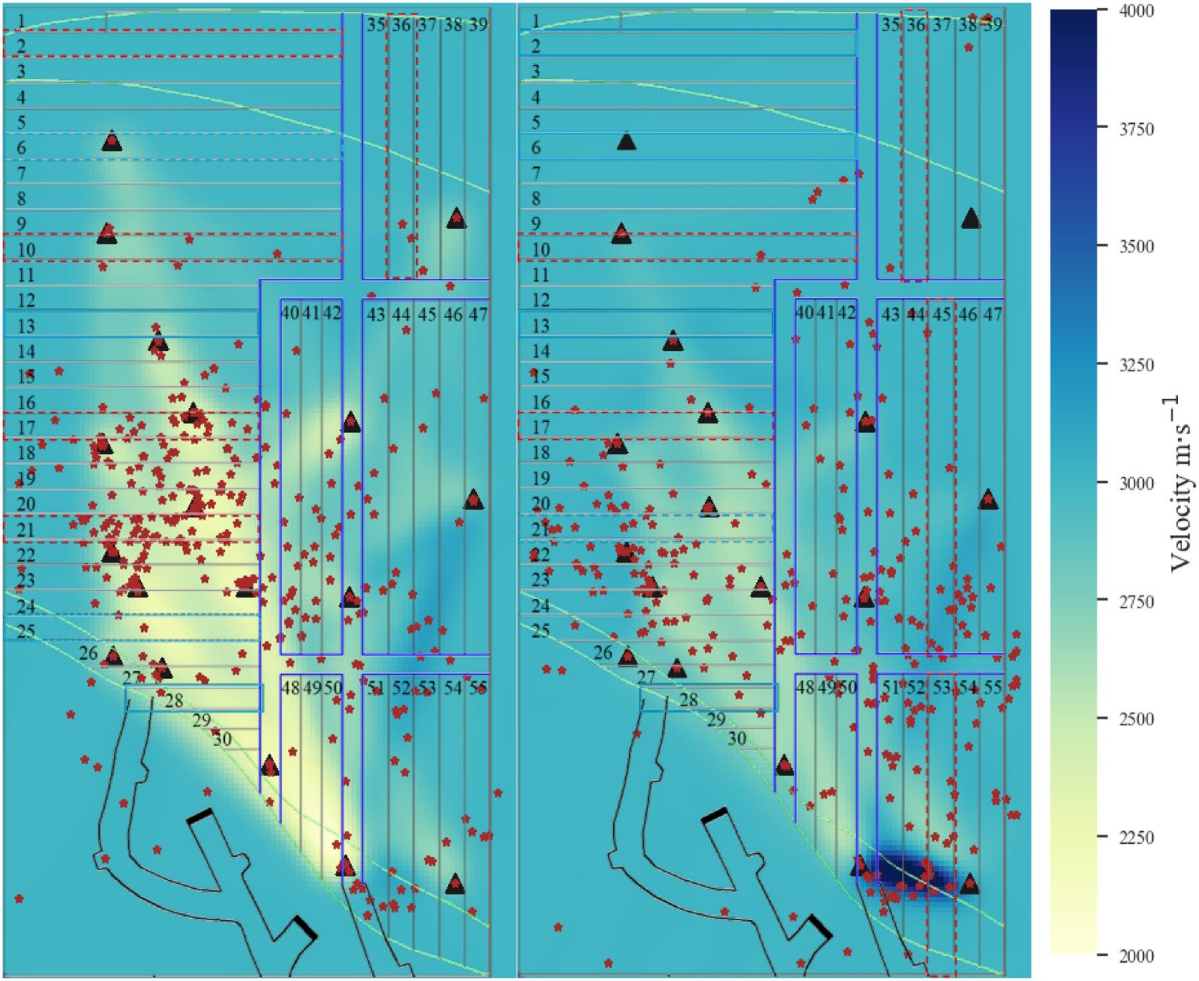
Tomographic imaging of the velocity field in the VI panel stope of the 1018 m sublevel. (**a**) From March 3, 2023 to March 11, 2023. (**b**) From March 17, 2023, to March 27, 2023.

As shown in Fig. 6a, production activities such as blasting and mining operations in the 17# and 21# drifts and backfilling operations in the 25# and 28# drifts were performed between March 3, 2023, and March 11, 2023, which significantly affected the velocity in the production area. The quantity of the microseismic events used for tomography, between March 3, 2023, and March 11, 2023, was 736. This resulted in a large area of low velocity, bounded by the 17# drift and the ore-rock interface zone, which corresponds to the nucleation area of microseismic events. The formation of this low-velocity area is related to three main factors. First, the overlying roof of the production drifts experiences stress release during the blasting and excavation phase, accompanied by subsidence and deformation of the filling body. Second, the filling body in the 25# and 28# drifts may exist in a semiliquid or even unconsolidated state, resulting in significant acoustic wave diffraction. Third, the blasting activities cause damage to the nearby filling and ore body, creating fractures and localized fragmentation. Furthermore, the velocity increases slightly at the intersection between the cross drift and the connection roadway located in the ore-rock interface zone. In this area, there is also a concentration of microseismic events , which may be attributed to stress transfer from the overlying fill body roof to these special structures due to frequent stress changes in the mining area. In addition, the velocity in the area in which the ore body has not yet been excavated or has been backfilled remains close to 3000 m/s, indicating the overall stability of the fill body roof.

The changes in the velocity field of the roof between March 17, 2023, and March 27, 2023, were significantly different from those during early March (Fig. 6b). The quantity of the microseismic events used for tomography, between March 17, 2023, and March 27, 2023, was 623. The velocity noticeably increased in the areas previously affected by blasting and mining operations, indicating that as the curing time increased, the strength of the backfill in the drifts gradually increased. Moreover, the lower-layer backfill provides new support for the upper-layer backfill, leading to a reduction in stress relaxation and roof creep. Additionally, the velocity increases significantly at the intersection between the 1# cross drift and the connection roadway. In this area, there is also a concentration and nucleation of  microseismic events, intensifying the stress concentration in the ore-rock transition zone at the hanging wall.

**Figure 7:**
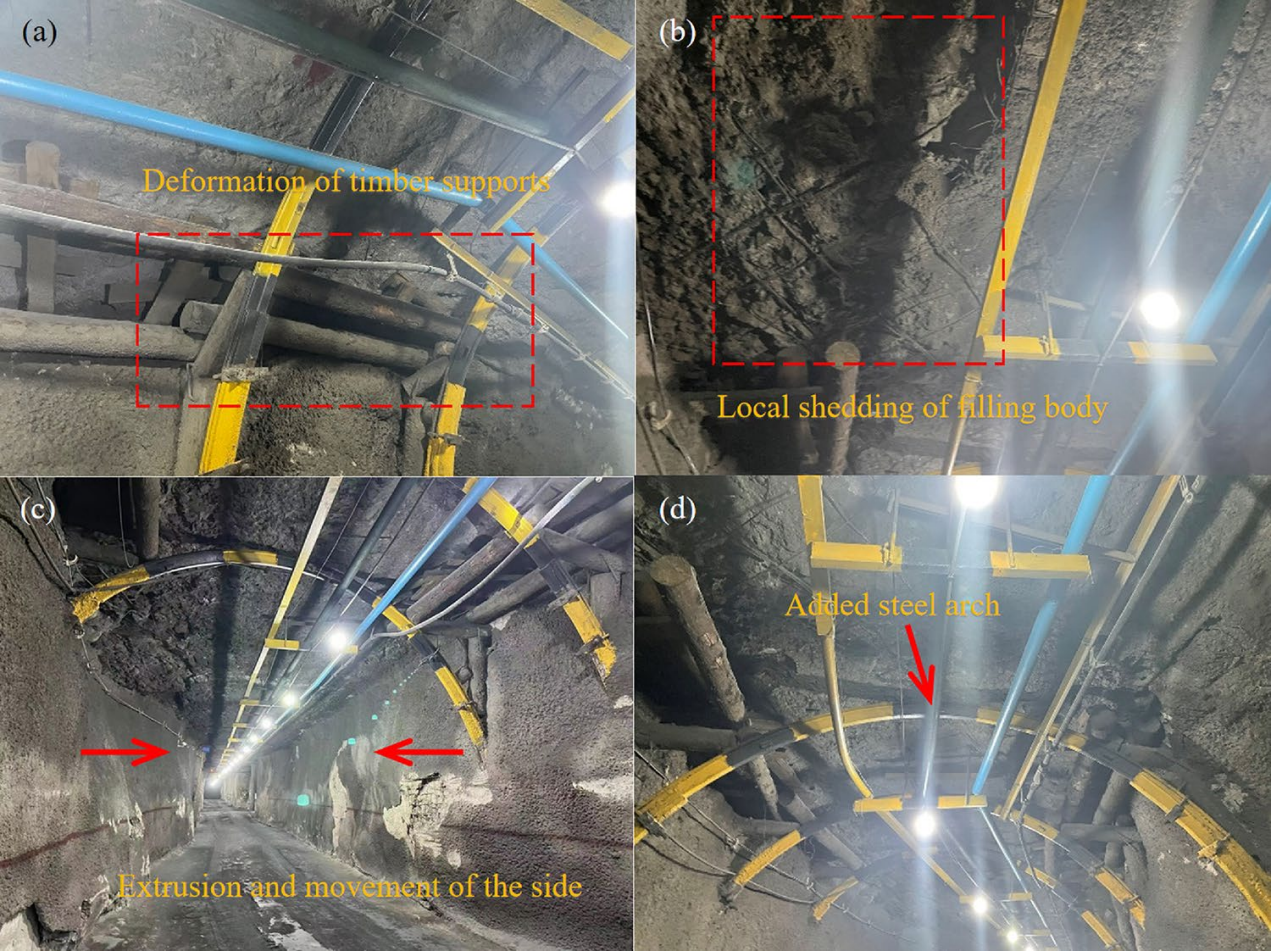
Deformation and damage of the intersection between the 1# cross drift and the connection roadway located in the ore-rock interface zone on April 13, 2023. (**a**) Supporting structures for the layer connection roadway outside the vein in the transition zone. (**b**) The fill body roof within the vein in the transition zone. (**c**) Failure of the roadway side wall caused by stress release. (**d**) Recently added steel arch to increase the support area.

Figure 7 shows a photo taken at the connection area on April 13, 2023. The transition area between the rectangular drift and the arched roadway is supported by a steel arch frame, and wood is used on both sides of the arch frame to connect different parts of the roof. Due to stress concentration, the circular timber columns and steel arch show severe compression and deformation. Moreover, tensile stress caused the surrounding fill body roof to detach from the structure, resulting in a certain degree of fragmentation. This is consistent with the high-velocity area observed in the imaging analysis, indicating a high degree of stress concentration in the mineral rocks and backfill in this area.

**Figure 8:**
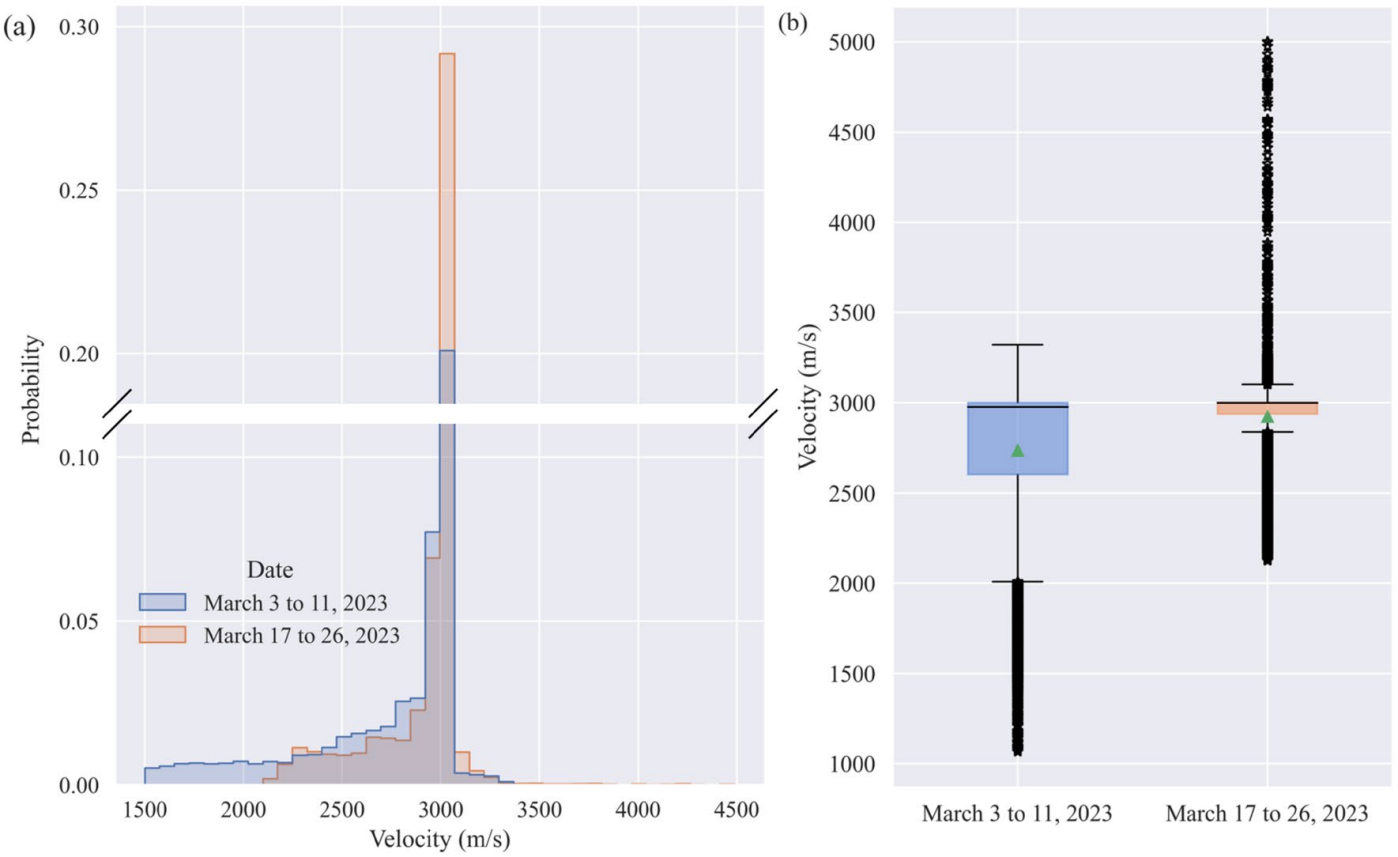
Statistical comparison of the wave velocity distribution. (**a**) Frequency histogram; (**b**) Box diagram (the triangles represent the mean, and the black lines represent quantiles).

Furthermore, according to the wave velocity distribution statistics of the grid nodes in the panel mining area, the average and median wave velocities for the periods March 3 to 11 and March 17 to 27 were 2736 m/s and 2976 m/s (Interquartile range: 2603 m/s, 3000 m/s) and 2921 m/s and 2999 m/s (Interquartile range: 2935 m/s, 3001 m/s), respectively (Fig. 8). The wave velocities at the grid nodes within the panel mining area in March are primarily in the range of 2600 m/s to 3000 m/s, which are close to the average results obtained from the wave velocity on-site sampling measurement and the wave velocity inversion analysis based on the geoacoustic event rays. Moreover, the wave velocities within this range accounted for approximately 50% of the overall distribution, indicating that the fill body roof is complete and stable. However, comparing the data for March 3 to 11 with that for March 17 to 27, significant changes can be observed in localized areas, marked by three key variations: 1. The minimum wave velocity increased from 1065 to 2126 m/s, the node wave velocities below 2600 m/s decreased significantly, and the low-velocity areas below 2000 m/s disappeared. This is attributed to the substantial reduction in the goaf area inside the panel mining area and the controlled behavior of the original exposed roof area. 2. A significant number of nodes have wave velocities of approximately 3000 m/s, and the standard deviation decreased from 419 to 251 m/s, indicating a decrease in the anisotropy of the wave velocity field within the panel mining area. 3. The number of nodes with wave velocities exceeding 3000 m/s increased. The maximum wave velocity from March 3 to 11 was 3321 m/s, while a substantial number of nodes with wave velocities exceeding 4000 m/s were observed from March 17 to 27. This suggests that the stress concentration was exacerbated in the original high-velocity area.

### Spatial *b*-value scanning

**Figure 9:**
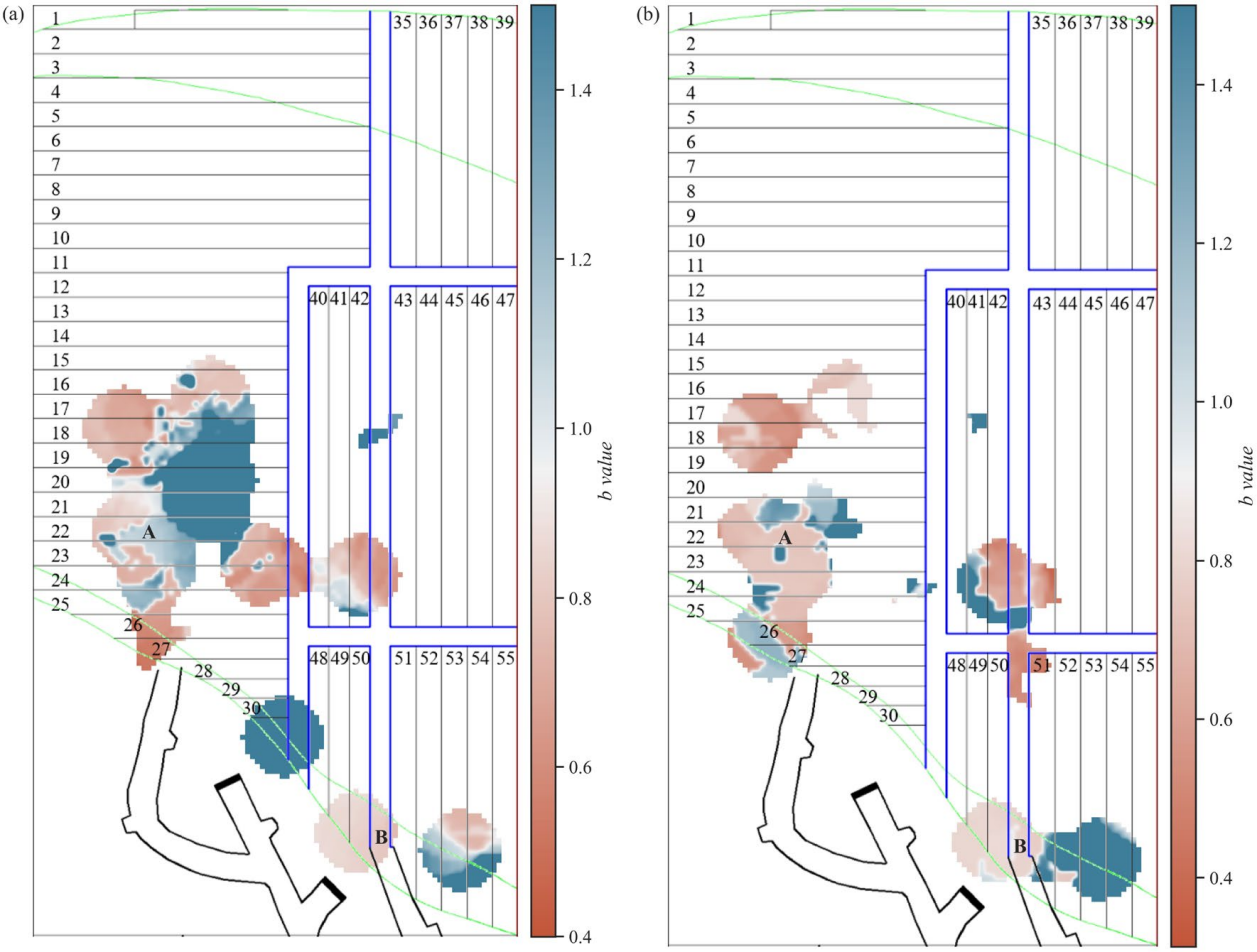
Spatial *b*-value scanning in the 6 mining zones of the 1018 m intervals. (**a**) From March 3, 2023 to March 11, 2023. (**b**) From March 17, 2023, to March 27, 2023.

**Figure 10:**
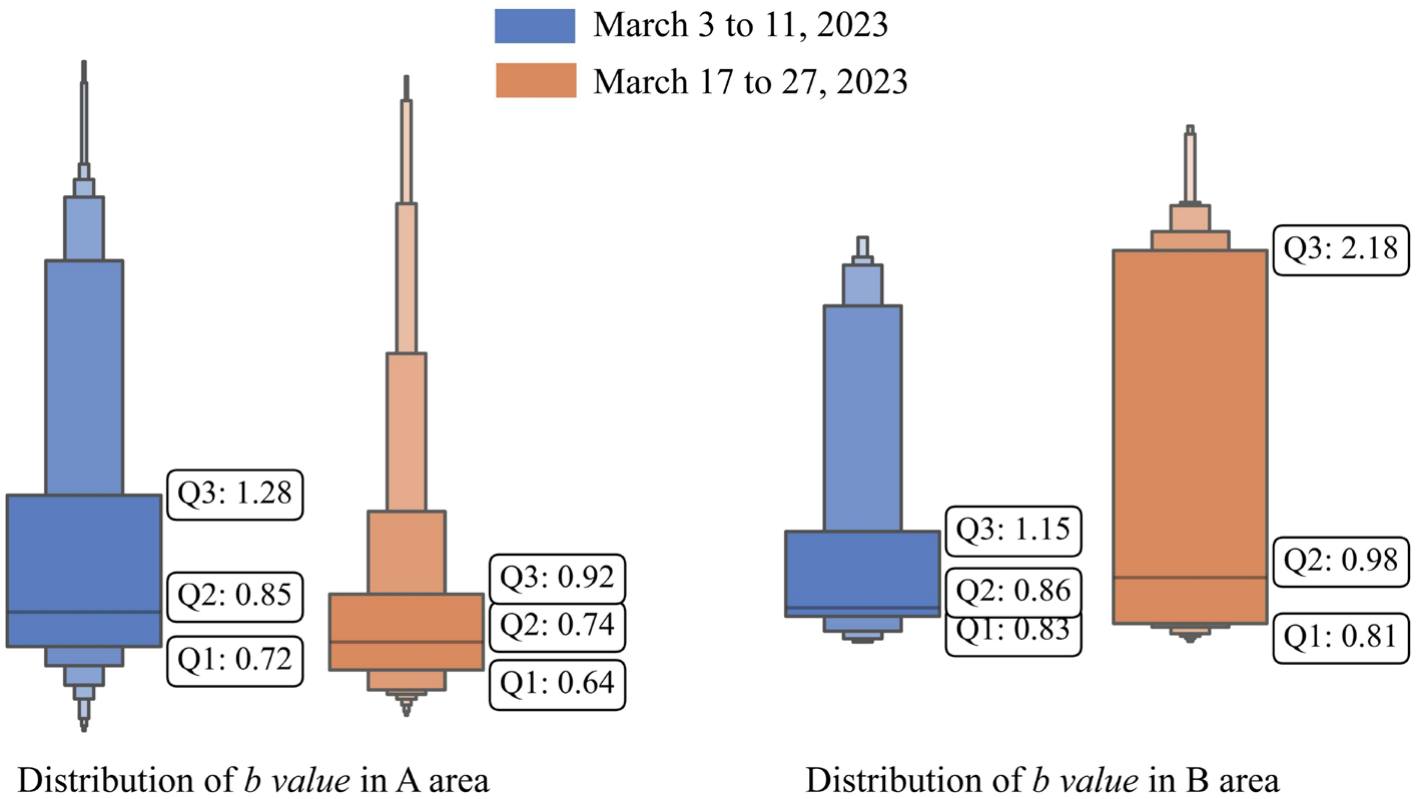
Statistical comparison of the *b-value* distribution in different areas (corresponding to the areas marked in Fig. 9).

Mining-induced seismicity and crustal earthquakes have different scales, but they have similar intrinsic mechanisms. Generally, the *b*-value fluctuates around 1.0. The *b*-value comprehensively reflects the rock properties, stress state, and structural characteristics in the area. A lower *b*-value corresponds to increased stress concentration and increased seismic activity. The spatial distribution of the *b*-value in the panel stope in March 2023, which was influenced by mining operations performed in the area, exhibits strong heterogeneity and effectively reflects the transfer of the stress state (Fig. 9). Both the directly affected area A and the indirectly affected area B affected by mining activities exhibit extensive *b*-value changes. The spatial b-value distribution within these areas is shown in Fig. 10.

The low *b*-value area aligns with the high-risk area where the stress changes significantly due to the mining operations. This area mainly includes the intersection areas between the cross drift and the connection roadway, the areas close to the blasting face in the mining drift, the drifts with prolonged roof exposure, and some drifts adjacent to the mining areas. These areas may have strong seismic activity when the *b*-value is below 0.8. The relationship between low *b*-values and the concentration and release of stress in the area depends on an understanding of the production activities and the structural characteristics of the mining field. On the one hand, the mining activities in drifts 17# and 21#, as well as the blasting operations in the 5th layer, lead to stress release in the overlying fill body roof and damage to the surrounding ore bodies and backfill area. The lower quartile of the spatial *b*-value for mining-induced microseismic activity during the two periods are 0.72 and 0.64, respectively, forming low *b*-value areas accompanied by the nucleation of microseismic events. On the other hand, specific structures within the stope, such as the ore body contact zones, intersections among connection roadways, and cross/strike drifts, are susceptible to frequent mining-induced stress redistributions. These areas are considered semipermanent drifts, and assuming that the stope structure remains unchanged, it is evident that stress changes primarily occur within the roof and rock. From March 17 to March 27, the mining activities in the 45# and 53# drifts lead to the formation of new low *b*-value zones near the intersection of the 1# cross drift and 1# strike drift, accompanied by a slight increase in wave velocity. Especially in the anomalous *b*-value zones at the intersection of the connection roadway and the strike drifts, the *b*-values are higher than the spatial *b*-values in the mining areas. The lower quartile of the spatial *b*-values in these regions is around 0.8, indicating a higher proportion of large-scale microseismic events. This suggests a gradual release of stress, which corresponds to the observed damage in the support structures and fill body roof over time.

Seismic activity is often associated with aftershock sequences following the main shock in high *b*-value areas. In parts of the 17# and 21# mining drifts, which are farther from the blasting face and have longer cessation times, the *b*-values typically exceed 1.0. According to Fig. 10, by late March, the upper quartile of the spatial *b*-value decreased from 1.28 to 0.92, and the high *b*-value regions disappeared with the cessation of production. Additionally, the number of seismic sources decreased, and smaller-magnitude events dominate the seismicity distribution. This indicates that after the stress relaxation in the roof of the backfill body, the system enters an adjustment or creep state, with a relatively low stress level. In contrast, the high *b*-value area might indicate stress accumulation and potential future stress release. In late March, the median and upper quartile of the spatial *b*-values in area B reached 0.98 and 2.18, respectively, experiencing numerous small-scale microseismic events that failed to effectively release sufficient stress. Over time, this accumulation may lead to larger-scale rock failure activity, which corresponds to the roof damage observed in this area in April.

## Discussion

### The stress migration patterns in the potential high-risk destabilization areas

**Figure 11:**
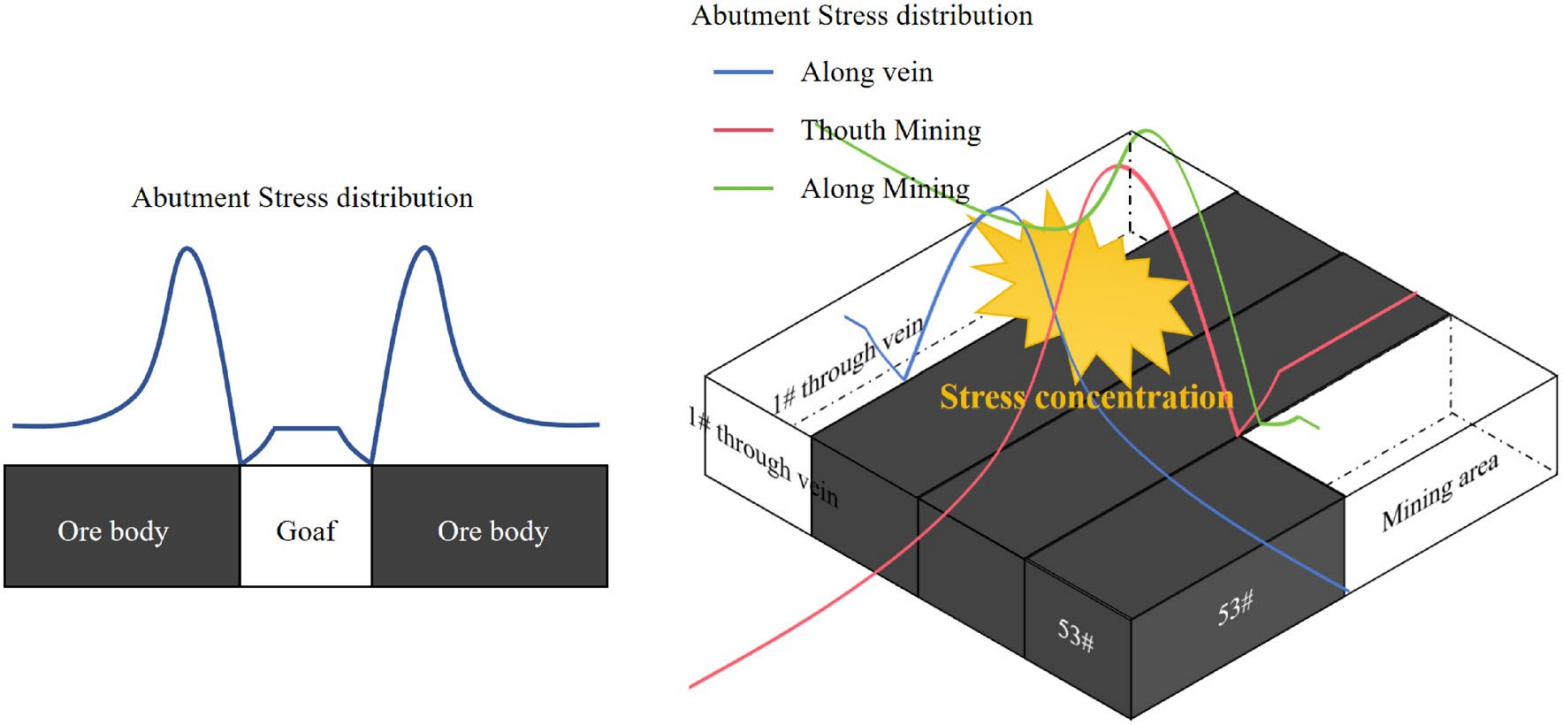
Schematic diagram of the advanced abutment stress redistribution in the roof under mining influence.

The stress redistribution process in the panel stope can be explained by the advanced abutment stress distribution model of the rectangular roadway roof considering the influence of mining activities (Fig. 11). In the drift and fill extraction method, the loading of the fill body roof in the extraction area is transmitted to the adjacent areas, showing an initial increase followed by a gradual decrease as the distance from the source increases. Moreover, the tensile stress increases at the bottom of the fill body roof, and compressive stress areas appear on both sides of the drift. As a result, abnormal deformation and damage to the surrounding backfill area and ore body are more likely to occur near extraction drifts. Furthermore, the advanced abutment stress induced by mining activities and the residual abutment stress in the originally excavated semipermanent drifts (such as the cross drifts, strike drifts, and connection roadway) should both be considered. At this stage, the peak supporting stress is likely to be generated in the direction of the blasting face or in the surrounding areas of the semipermanent drifts. Importantly, stress changes and their potential impact areas are highly complex and are influenced by many factors, including stope engineering, geological structures, physical properties of ore bodies, and backfill materials. The presence of unique structural and construction zones increases the potential for stress concentrations in surrounding ore bodies and backfill areas. Specifically, three areas with significant stress changes exist.

#### The goaf area near the blasting face in the drifts

When the drift and fill extraction method is applied in the panel stope, the original stress field between the upper filling body and the ore body is disrupted. The self-weight stress of the upper backfill in the goaf area is transferred to new support points near the adjacent backfill drift and ore body, resulting in elevated stress concentrations. Simultaneously, the exposed fill body roof is subjected to significant tensile stress. Additionally, as the blasting face of the drift continuously advances, the stress in the newly excavated areas is instantaneously released, and dynamic stress continuously acts on the adjacent areas with abnormal stress distributions. Blast disturbances can also affect the impact damage to the backfill body and ore body located on the roof and two sides of the drift. As a result, safety incidents such as side collapses and roof falls frequently occur within extraction drifts. This coincides with the occurrence of nucleation microseismic events, significant decreases in velocity field, and low *b*-value zones near the blasting face. As the mined-out area in the drift gradually moves away from the blasting face, the influence of mining activities diminishes, and the behavior of the backfill roof stabilizes, entering a slow creeping state. The frequency and intensity of the microseismic activity also decrease, and the low *b*-value zones become high *b*-value zones.

#### Semipermanent drifts adjacent to the mining drifts

The stress distribution in the strike drift and cross drift of the panel stope tends to be stable after the initial excavation operations. The roof of the overlying fill body in the exposed area undergoes long-term creep deformation under a constant load, accompanied by a low settlement deformation rate^23,24^. However, when affected by nearby blasting excavation activities, the residual abutment stress from the upper layers and original abutment stress both impact the area, leading to repeated redistribution of stress. The dynamic disturbance caused by blasting triggers an instantaneous response of the exposed roof and may hasten its creep deformation under creep stress. This situation repeats after the next dynamic disturbance, potentially leading to instability in the roof, especially in areas with poor filling quality. This results in the initiation of some microseismic activity, along with a decrease in the wave velocity in the fill roof area and the appearance of low *b*-value zones.

#### The ore-rock transition zone and the intersection between the connection roadway and the cross drift

In the Jinchuan No. 2 mining area, the external veins consist of fragmented rocks formed by frequent intrusion and intercalation of various magmatic rocks. It has a variable crystalline block structure. Weak structural planes are formed between the peripheral rocks, ore bodies and filling bodies at the hanging wall^25^. Furthermore, the intersection between the connection roadway and cross drift is an engineering structure in which rectangular tunnels and straight-wall arched tunnels intersect, forming an angle that deviates toward the 53# roadway. Under the influence of mining activities, stress tends to migrate toward this area with unique engineering and geological conditions. By late March, as the 53# drift excavation approached the hanging wall, high and low *b*-value zones appeared simultaneously within the high wave velocity area, accompanied by relatively strong microseismic activity. Moreover, larger seismic events may occur in this area. Notably, the high *b*-value zones and high wave velocity zones, as well as the location of the fill roof collapse in Fig. 6b, are all skewed toward the side of the 53# production drift. This may mean that the simultaneous occurrence of high *b*-value and high wave velocity zones is related to the continued accumulation of energy and increased anisotropy in the area.

### Advanced risk area identification method using collaborative spatial *b*-value scanning and wave velocity field tomography

The imaging accuracy varies spatially due to the nonuniform coverage of the monitoring networks. In addition, the finite difference method assumes continuous changes in wave velocity, which results in the wave velocity field reflecting the relative stress evolution in a larger range in the target area. To address this limitation, spatial variations in *b*-values within the area can be used to more accurately identify stress characteristics in localized areas and better explain changes in the stress gradients. This comprehensive method uses spatial *b*-value scanning to identify areas with anomalous microseismic activity. Low *b*-value areas indicate enhanced microseismic activity and stress release, while high *b*-value areas reflect stress adjustment or continued stress accumulation. Moreover, the seismic velocity field tomography provides a means to gain a deeper understanding of changes in the stope structure and the quality of the ore body and rock mass. By analyzing the velocity data and variations, areas where mining structure integrity is compromised or with significantly increased stress can be identified. Combining data analysis from the panel stope and observations triggered by mining activities, it can be inferred that: the low *b*-values and significant velocity gradient changes within an area can explain the stress release and concentration caused by mining activities, and areas with both high *b*-values and increased wave velocity may indicate the potential for severe hazards in the future. Overall, through the collaborative application of spatial *b*-value scanning and seismic velocity field tomography, areas in complex mining environments with potential safety issues can be identified and predicted. This allows mining and supporting operations to proactively address hazardous areas by applying timely support measures and optimizing mining plans.

## Conclusion

This study proposes a synergistic approach that integrates multisource wave velocity tomography imaging, utilizing both active and passive sources, with maximum likelihood estimation through spatial *b*-value scanning. This combined methodology aims to monitor seismic activities and proactively identify potential instabilities in mining operations caused by downward drifts and fill stoping. Combined with the space–time–magnitude distribution characteristics of the seismic signals, the stress migration and evolution laws of the stope structure during mining operations were analyzed. The three main forms of stress activities in the pillar-free large-area continuous stope are stress release of the drifts and subsidence of the fill roof after excavation, the stress redistribution of semipermanent drifts in adjacent areas caused by mining excavation operations, and increased stress concentration in the intersection area between special engineering structures and geological bodies. Furthermore, the high-risk areas directly induced by mining activities during stress migration exhibit significant low *b*-value and low wave velocity characteristics, while the high *b*-value characteristics in adjacent areas may reflect aftershock activity following the initial shock. Anomalous spatial *b*-values and high wave velocity regions correspond to increased stress concentration in non-extraction areas. Under this premise, high *b*-values and microseismic events nucleation indicate the potential for intense future stress release. The proposed method can enhance the understanding of the evolution mechanism of underground stress due to mining activities, enabling the advanced identification of high-risk, unstable areas. This study can be extended to more panel production conditions, utilizing big data to further quantify trends and thresholds of indicators under similar geological and mining conditions. Combining source mechanism analysis with attenuation inversion methods, the types and risk levels of instability hazards can be assessed to establish precise prevention and control measures.

## Data Availability

The authors will supply the relevant data in response to reasonable requests. For data requests, please contact Zhongwei Pei at the Email: csupeizw@csu.edu.cn.
